# Integrated Metabolomic Profiling and Harvest Volatile Signatures Reveal Cultivar-Specific Quality Traits in Blueberries (*Vaccinium corymbosum* L.)

**DOI:** 10.3390/plants15060948

**Published:** 2026-03-19

**Authors:** Marina-Rafailia Kyrou, Dimos Stouris, Athanasios Besis, Fokion Papathanasiou, Evangelos Karagiannis

**Affiliations:** 1 Department of Agriculture, School of Agricultural Sciences, University of Western Macedonia, 53100 Florina, Greece; 2 Environmental Pollution Control Laboratory, Department of Chemistry, Aristotle University of Thessaloniki, 54124 Thessaloniki, Greece

**Keywords:** *Vaccinium* spp., aroma compounds, primary metabolites, anthocyanins, phenolics, antioxidant capacity, postharvest

## Abstract

Blueberries (*Vaccinium corymbosum* L.) are widely appreciated for their flavor, bioactive compounds, and health promoting properties, yet cultivar-dependent differences in metabolic composition and postharvest stability remain incompletely understood. This study evaluated five commercial blueberry cultivars (‘Aurora’, ‘Chandler’, ‘Elliot’, ‘Legacy’, and ‘Liberty’) at harvest and after 15 days of cold storage (postharvest stage) (4 °C), assessing fruit color, size, firmness, primary metabolites, volatile organic compounds (VOCs), anthocyanins, phenolics, and antioxidant capacity. Cultivar-specific differences were observed in fruit morphology, sugar/acid balance, and biochemical composition: ‘Liberty’ and ‘Elliot’ accumulated higher monosaccharides and disaccharides, whereas ‘Aurora’ and ‘Chandler’ showed higher organic acids and amino acids. Volatile profiling at harvest revealed that ‘Liberty’ exhibited the richest aromatic profile, with elevated aldehydes, ketones, acids, phenols, alcohols, and esters. Postharvest storage caused minor changes in primary metabolites but altered anthocyanin content in a cultivar-dependent manner. Principal component analysis indicated that volatile compounds were the primary factors differentiating cultivars, while primary metabolites largely influenced sweetness–acidity balance. Overall, the results demonstrate that blueberry fruit quality is strongly cultivar-dependent, with cultivar-specific metabolic and volatile signatures shaping sensory and nutritional attributes, and provide valuable information for breeding, postharvest management, and cultivar selection to optimize flavor, bioactive content, and shelf-life.

## 1. Introduction

Blueberries (*Vaccinium corymbosum* L.) are globally recognized for their nutritional and economic importance, owing to their appealing sensory attributes and health-promoting bioactive compounds. These fruits are characterized by high concentrations of anthocyanins, polyphenols, and antioxidants [1,2,3,4,5]. Additionally, blueberries are valued for their specific sugar–acid balance and characteristic aroma, both of which are primary determinants of consumer acceptance and marketability [6,7].

The composition and quality of blueberries are influenced by both genetic factors, such as cultivar, and environmental conditions, including climate, soil, and agronomic practices [8,9,10]. Primary metabolites, including sugars, organic acids, and amino acids, define fruit taste, metabolic precursors, and nutritional quality, while volatile organic compounds are the main contributors to fruit aroma and perceived flavor intensity [11,12]. Recent studies have begun to unravel the complex metabolic networks defining blueberry quality. Specific sugar transporters and carbohydrate metabolism genes are differentially expressed among cultivars, directly impacting soluble solids content (SSC) and fruit firmness [9]. Major genetic loci controlling organic acid content have been identified, highlighting that acidity is a highly heritable trait that significantly alters the perception of sweetness [8]. The volatile landscape of numerous blueberry accessions has also been profiled, resulting in the categorization of distinct “aromatic types” based on the prevalence of particular terpenes and aldehydes [7]. However, while these traits vary significantly among cultivars, the relative contribution of primary versus secondary metabolites to flavor perception and their coordinated stability during postharvest storage remains a subject of ongoing debate [13]. Postharvest storage can substantially impact blueberry quality, particularly anthocyanin content, firmness, and aroma composition [14]. While sugars and organic acids often remain relatively stable, volatile compounds and phenolics may degrade during cold storage (postharvest stage), leading to reductions in sensory appeal and nutritional value [15,16]. Previous treatments, such as the use of photoselective nets during plant cultivation, have been employed to improve shelf-life by reducing moisture loss and pigment degradation; however, their effectiveness is highly cultivar-dependent, reflecting complex interactions between cultivar and storage conditions [17].

Despite increasing research, studies that simultaneously integrate primary metabolites, volatile profiles, and biochemical traits across multiple blueberry cultivars remain limited. This creates a gap in our understanding of how these metabolic networks collectively shape sensory quality and nutritional value [6]. Furthermore, there is limited information on how these primary and volatile metabolic components are jointly affected by postharvest cold storage (postharvest stage) across diverse cultivars. The present study aims to comprehensively evaluate five commercially significant blueberry cultivars (‘Aurora’, ‘Chandler’, ‘Elliot’, ‘Legacy’, and ‘Liberty’) at harvest and following cold storage (postharvest stage). By integrating analyses of color, physical attributes, primary metabolites, volatiles, and bioactive compounds, we sought to: (i) characterize cultivar-specific metabolic signatures and define the maximum aromatic potential of each cultivar at harvest; (ii) assess the postharvest stability of key physicochemical traits, primary metabolites, and bioactive compounds during cold storage; and (iii) identify the principal metabolites and volatiles driving cultivar differentiation. Multivariate analyses, including Principal Component Analysis (PCA), were employed to reveal the integrated metabolic signatures associated with fruit quality. We hypothesize that the cultivar (genotype) exerts a dominant effect over the storage period in determining the metabolic and aromatic profiles of highbush blueberries. Volatile profiling was performed at harvest to characterize cultivar-specific aromatic fingerprints, whereas postharvest effects were evaluated for physicochemical traits, primary metabolites, and bioactive compounds.

## 2. Materials and Methods

### 2.1. Plant Material and Experimental Design

Fruits of five northern highbush blueberry (*Vaccinium corymbosum* L.) cultivars (‘Aurora’, ‘Chandler’, ‘Elliot’, ‘Legacy’, and ‘Liberty’) were hand-harvested in July 2023 at full maturity, defined by uniform blue coloration according to commercial standards. Fruit physiological maturity was further characterized by measuring soluble solids content (SSC) and titratable acidity (TA) at harvest (day 0). All fruits were harvested and transferred to the laboratory within one hour for analysis. The experiment was conducted in a commercial orchard located in Amindeon, Western Macedonia, Greece. The plants were 7 years old, spaced at 3 × 1.2 m, and managed under standard horticultural practices, including drip irrigation, fertilization, pruning, and pest and disease management according to regional commercial recommendations. Climatic conditions during the 2023 growing season were typical for the region and did not show major deviations from multi-year averages.

A total of 240 fruits per cultivar were collected, with 120 fruits allocated to the ‘harvest’ stage and 120 to the ‘postharvest’ stage. For the postharvest evaluation, fruits were stored without precooling under standard commercial cold-storage conditions at 4 °C and 90–95% relative humidity for 15 days under ambient atmosphere and absence of packaging. The 15-day cold storage (postharvest stage) period was selected to simulate a realistic commercial postharvest timeframe for fresh blueberries, covering typical handling, transportation, and short-term retail distribution. For each cultivar, the 120 fruits per stage were distributed as follows: 30 fruits for morphological and physicochemical measurements (fruit weight, dimensions, color traits, flesh firmness, soluble solids, and titratable acidity); 30 fruits for primary metabolomic analysis; 30 fruits for volatile organic compounds (VOCs) analysis; and 30 fruits for biochemical analyses (total antioxidant capacity, total phenolic content, and total anthocyanin content). Each category was divided into three biological replicates, consisting of 10 fruits per replicate. All fruit samples were immediately frozen and stored at −80 °C until further analysis.

### 2.2. Fruit Quality Assessment

#### 2.2.1. Color Measurement

Blueberry fruits were assessed with the natural wax intact. Color parameters (L*, a*, b*) were recorded using a colorimeter (model CR-410, Konica Minolta, Osaka, Japan) equipped with a D65 illuminant and a 2° standard observer. Measurements were performed in contact mode by placing the fruit surface directly against the instrument’s 50 mm measuring aperture (*φ*). The 60 mm light projection tube was removed to allow direct contact between the fruit and the sensor. The instrument was calibrated using the manufacturer’s white calibration plate before use. Chroma (C*) and hue angle (*h*°) were calculated as follows: C* = √[(a*)^2^ + (b*)^2^] and *h*° = arc tan (b*/a*) [18]. Measurements were taken on ten fruits per replicate for each cultivar.

#### 2.2.2. Fruit Attributes

The fresh weight (FW) of each fruit was measured using a precision balance (Shanghai Sunny Hengping Electronic Precision Balance, model JA4103, Shanghai, China). Fruit dimensions were recorded in millimeters (mm) using a digital caliper, with measurements taken along the two principal axes, width and height.

#### 2.2.3. Soluble Solids Concentration and Titratable Acidity

The soluble solids concentration (SSC, %) and titratable acidity (TA, % malic acid equivalents) of the blueberry fruits were determined using a portable electronic refractometer (Atago Co. Ltd., Tokyo, Japan) and an automatic titrator (HI 84532, Hanna Instruments, Woonsocket, RI, USA), respectively [19]. Juice was extracted from freshly harvested fruits and filtered prior to analysis. SSC was measured at room temperature and expressed as a percentage, while TA was determined by titration with a standardized NaOH solution (0.1 N) and expressed on a fresh weight basis (%, FW). Moreover, the SSC/TA ratio was calculated by dividing SSC by TA.

#### 2.2.4. Textural Properties

Fruit firmness was determined by measuring the maximum penetration force of the peel at two opposing sites of each fruit using a texture analyzer (Model GS-15, GUSS, Strand, South Africa). The instrument was equipped with a 2 mm diameter flat-end needle probe. Measurements were conducted at a penetration speed of 5 mm s^−1^ to a final depth of 4 mm.

### 2.3. Determination of Total Phenolic Content and Antioxidant Capacity

The extraction of polyphenolic substances was performed according to Karagiannis et al. [20]. One gram of fruit was homogenized with 25 mL of extraction solution (acetone:water:acetic acid, 70:29.5:0.5, *v*/*v*/*v*). The solution was kept at 4 °C in the dark for 48 h; after centrifugation (10,000× *g*, 4 °C, 10 min) an aliquot of the supernatant was used for the analysis. Total phenols were determined following Folin–Ciocalteu method at 760 nm [21], with results expressed as mg gallic acid equivalents g^−1^ fresh weight (FW). The 2,2′-azino-bis (3-ethylbenzthiazoline-6-sulphonic acid) (ABTS) free radical-scavenging activity was assessed as described by Karagiannis et al. [20]. The antioxidant potential of the extracts was determined using Trolox as the standard and the results were expressed as μg Trolox equivalents g^−1^ fresh weight (FW).

### 2.4. Determination of Total Anthocyanin Content

Total anthocyanin content was estimated by the pH-differential assay (AOAC method 2005.02) with slight modifications [20]. One (1) gram of frozen tissue was homogenized in 10 mL of extraction solution (ethanol:water:HCl, 80:19:1, *v*/*v*/*v*). Frozen tissue consisted of homogenized whole berries (skin and flesh), and the same extraction protocol was applied to all cultivars and storage stages to ensure consistency. The solution was kept in the dark at 5 °C for 24 h and then centrifuged (10,000× *g*, 4 °C, 10 min) and the supernatant was collected; the procedure was repeated, and the final volume was adjusted to 25 mL. Quantitative determination was accomplished in a spectrophotometer (SPECORD 210 Plus, Analytik, Jena, Germany) by measuring the absorbance at 515 nm and 700 nm. Samples were adjusted to pH 1.0 (with potassium chloride buffer 0.02 M) and to pH 4.5 (with sodium acetate buffer 0.4 M). After pH adjustment, all samples were brought to the same final volume. The results were expressed in mg cyanidin equivalents 100 g^−1^ fresh weight (FW).

### 2.5. Primary Metabolite Profiling

Primary polar metabolites were extracted from the fruit as described in [22] at harvest and after cold storage (postharvest stage) stages (0 d at 20 °C). Frozen lyophilized tissues (0.5 g) were transferred into 15 mL falcon tubes, and 1.4 mL of precooled (−20 °C) pure methanol was added. An internal quantitative standard (adonitol, 100 μL of 0.2 mg mL^−1^) was then added, and the samples were incubated for 10 min at 70 °C. After centrifugation (11,000× *g*, 4 °C, 10 min), the supernatant was collected, and 750 μL of precooled (−20 °C) chloroform and 1500 μL of cold (4 °C) dH_2_O were added. The mixture was centrifuged again (2200× *g*, 4 °C, 10 min), and the upper polar phase was transferred to 1.5 mL glass vials and evaporated to dryness under a gentle nitrogen stream. The dried extracts were derivatized by dissolving them in 40 μL of methoxyamine hydrochloride (20 mg mL^−1^) at 37 °C for 2 h under gentle shaking, followed by the addition of 70 μL of N-methyl-N-(trimethylsilyl)trifluoroacetamide (MSTFA) and incubation for 30 min at 37 °C.

GC–MS analysis was performed using a Shimadzu GC/MS-QP2010 system equipped with an AOC-20i autosampler (Shimadzu, Kyoto, Japan). One microliter of each sample was injected with a split ratio of 70:1 onto a 30 m × 0.25 mm × 0.25 μm Agilent capillary column. The injector temperature was set to 220 °C, the ion source to 230 °C, and the interface to 250 °C. The carrier gas was maintained at a constant flow of 1 mL min^−1^. The GC temperature program was as follows: initial temperature of 70 °C for 5 min, followed by an increase to 250 °C at 8 °C min^−1^, and held for 15 min. Mass spectra were recorded in EI mode over *m*/*z* 50–600 after a 3.5 min solvent delay. Data acquisition, peak integration, and chromatographic visualization were performed using GC/MS Solution software Ver. 2.4 (Shimadzu, Kyoto, Japan). The quantified metabolites were grouped into the following categories: monosaccharides (e.g., glucose, fructose), disaccharides (e.g., sucrose), polyols (e.g., sorbitol, mannitol), organic acids (e.g., citric, malic, quinic acids), amino acids (e.g., alanine, valine, serine), fatty acids (e.g., palmitic, stearic acids), lipids (β-sitosterol), and triterpenoids/sterols (e.g., oleamide). Identification was based on retention time and mass spectral matching with reference standards or NIST14/NIST17 libraries. Relative metabolite levels were quantified against the internal standard (adonitol) as described in [23]. Metabolite quantification was performed in a semi-quantitative manner relative to the internal standard, which is a common approach in GC-MS-based comparative metabolomics studies.

### 2.6. Volatile Organic Compounds (VOCs) Analysis

Sampling of volatile organic compounds (VOCs) from blueberry fruit was performed once at the commercial harvest stage (five cultivars, three biological replicates per cultivar), in order to capture the maximum aromatic potential of each cultivar. VOC analysis was therefore focused on cultivar-specific aromatic fingerprints at harvest, whereas postharvest changes were evaluated through physicochemical traits, primary metabolites, and bioactive compounds. Approximately 50 g of representative berries from each replicate were homogenized into pulp and immediately placed in a 5 L sealed glass chamber, where they were allowed to equilibrate for 1 h at room temperature. Air from the headspace was then drawn for 30 min at a constant flow rate of 100 mL min^−1^ using identical portable pumps (Gillian GILAIR-Plus Personal Air Sampling Pump, Sensidyne, St. Petersburg, FL, USA, 1–5000 cm^3^ min^−1^) connected to inert coated stainless-steel sorbent tubes (Markes International Ltd., Llantrisant, UK). The tubes were packed with a dual-bed combination of Tenax TA and Sulficarb, optimized for capturing a wide range of volatile and semi-volatile compounds including reactive sulfur species. Following sampling, the tubes were sealed with brass caps, transported to the laboratory in a clean, airtight refrigerated container (4 °C), and analyzed within 24 h. Thermal desorption–gas chromatography/mass spectrometry (TD-GC/MS) analysis was conducted using a UNITY-xr thermal desorption unit (Markes International Ltd., UK) coupled to a Shimadzu GC/MS-QP2020 system (Shimadzu, Japan). The analytical procedure followed the protocol [24] with minor modifications to accommodate the blueberry matrix. GC/MS conditions were optimized for the simultaneous quantification of 39 compounds, including 2 sulfides, 13 aldehydes, 2 ketones, 10 acids, 1 phenol, 6 alcohols and 5 esters. The quantification of VOCs was performed using the external standard method. For each of the 39 VOCs, four-point calibration curves were established, covering at least the full range of concentrations observed in real samples. The standard concentrations covered, at least, the range of concentrations encountered during the analysis of real samples. Calibration curves were tested daily. The Markes’ Calibration Solution Loading Rig (CSLR), specifically designed for loading sorbent tubes with liquid-phase standards, was used in the TD-GC/MS system. Standard solution was prepared so that the mass of analytes introduced in the injection volume matches the masses collected during in-cabin sampling. The liquid calibration standard (1.0 μL) was injected through the injector septum using a precision syringe, and onto the sampling end of the attached tube. The solution vaporized in the carrier gas flow (nitrogen, 50 mL min^−1^ for 3–5 min) and was passed onto the sorbent tube in the vapor phase. A sufficient volume of carrier gas was required to pass through the tube so that most of the carrier solvent (e.g., methanol) passes through the sorbent and away to vent while the compounds of interest are still quantitatively retained. Then, the tube was placed directly in the tube oven for analysis. Calibration curves were verified daily to ensure accuracy. The analyzed compounds exhibited repeatability of ≤20% (expressed as relative standard error, %RSE) (Supplementary Table S1). Limits of detection (LOD) were determined based on a signal-to-noise ratio of 3, while limits of quantification (LOQ) were defined as three times the corresponding LOD (Supplementary Table S1).

### 2.7. Statistical Analyses

Statistical analysis of physicochemical parameters and biochemical traits was performed using IBM SPSS Statistics (v29.0.2; IBM Corp., Armonk, NY, USA). To evaluate the effect of cultivar, a One-way Analysis of Variance (ANOVA) was conducted separately for each stage (harvest and post harvest). Because the study aimed primarily to evaluate cultivar-specific patterns within each stage rather than factorial treatment effects, storage was treated as a sequential evaluation stage rather than an independent experimental factor; therefore, cultivar comparisons were performed within each stage rather than using a factorial cultivar × storage model. Statistically significant differences among cultivars were detected using Duncan’s Multiple Range Test; *p* ≤ 0.05. Similarly, primary metabolites and VOCs were analyzed using SPSS, with significant differences determined using the LSD post hoc test (with * indicating *p* ≤ 0.05; ** indicating *p* ≤ 0.01; *** indicating *p* ≤ 0.001). Principal Component Analysis (PCA) was employed to visualize cultivar clustering based on metabolic profiles. For heatmap visualizations, ‘Legacy’ was used as the reference cultivar because it represents a well-established commercial genotype and exhibited intermediate values for most measured traits, facilitating the comparison of relative metabolite differences among cultivars.

## 3. Results

### 3.1. Color Parameters of Blueberry Cultivars at Harvest and Postharvest

This section analyzes the color parameters (L*, a*, b*, C*, and *h*°) of five northern highbush blueberry (*Vaccinium corymbosum* L.) cultivars (‘Aurora’, ‘Chandler’, ‘Elliot’, ‘Legacy’, and ‘Liberty’) at harvest and after 15 days of cold storage (postharvest stage). Measurements were taken to evaluate cultivar-specific coloration within each stage.

Regarding fruit lightness (L*), values at harvest were similar across cultivars, ranging from 11.2 to 12.5 (Table 1), with no significant differences observed among the evaluated cultivars. Although L* values decreased across all cultivars after 15 days of cold storage (postharvest stage), ‘Legacy’ and ‘Liberty’ retained significantly higher lightness compared to ‘Aurora’ and ‘Chandler’, while ‘Elliot’ exhibited intermediate values.

In terms of chromatic attributes at harvest, ‘Chandler’ exhibited the highest a*, b* and C* values. Conversely, ‘Chandler’ displayed the lowest hue angle, reflecting a more pronounced red-purple tonal component, whereas ‘Legacy’ and ‘Elliot’ showed higher *h*° values (more blue-green). ‘Liberty’ showed no significant difference in *h*° compared to ‘Chandler’ but had lower C* values. Following 15 days of cold storage (postharvest stage), ‘Chandler’ maintained higher a*, b* and C* values and lower *h*° compared to the other cultivars. This indicates that ‘Chandler’ fruit retained a more saturated coloration (higher C*) with more stable red and yellow chromatic contributions (a* and b*) during storage.

### 3.2. Fruit Quality Evaluation

The quality characteristics of five blueberry cultivars are examined, with reference to fresh weight (FW), dimensions (height and width), firmness, soluble solids content (SSC), titratable acidity (TA), and the SSC/TA ratio. Measurements were taken at harvest and after 15 days of cold storage at 4 °C, allowing for the assessment of cultivar-specific differences within each stage. This analysis provides insight into how varietal traits influence both the physical integrity and the chemical composition of the fruit during storage.

The qualitative attributes of five blueberry cultivars (‘Aurora’, ‘Chandler’, ‘Elliot’, ‘Legacy’, and ‘Liberty’) at harvest and after cold storage (postharvest stage) are presented in Table 2. At harvest, the cultivar ‘Chandler’ exhibited significantly higher fruit weight (1.74 g) and larger dimensions (height: 11.4 mm, width: 15.4 mm), confirming its characterization as a large-fruited cultivar. In contrast, ‘Legacy’, ‘Liberty’ and ‘Elliot’ showed the lowest fresh weight (1.13–1.24 g). Regarding fruit firmness, the highest values were recorded in ‘Legacy’ (68.2) and ‘Liberty’ (66.7), whereas ‘Elliot’ and ‘Aurora’ presented lower firmness values (56.2 and 62.8, respectively), suggesting cultivar-dependent differences in texture that may influence postharvest behavior. In terms of chemical composition, SSC values ranged from 11.5% to 14.2%. ‘Legacy’ exhibited the highest SSC (14.2%), followed by ‘Liberty’ (12.6%) and ‘Chandler’ (12.4%), while ‘Elliot’ and ‘Aurora’ showed lower values. TA varied from 0.7% to 2.1% (malic acid equivalents), with ‘Aurora’ exhibiting the highest acidity and ‘Legacy’ the lowest. Consequently, the SSC/TA ratio was highest in ‘Legacy’ (19.7) and lowest in ‘Aurora’ (5.8). This high ratio in ‘Legacy’ suggests a potentially sweeter taste perception, as indirectly inferred from the sugar–acid balance without direct sensory evaluation. Following cold storage (4 °C for 15 days), a significant difference in fruit firmness was observed between ‘Liberty’ (most firm) and ‘Elliot’, Chandler’ and ‘Aurora’ (less firm). SSC values remained stable, while TA decreased, resulting in higher SSC/TA ratios, particularly in ‘Legacy’ and ‘Liberty’. This observation is consistent with previous studies demonstrating that cultivar strongly influences fruit quality traits in many fruit crops.

### 3.3. Biochemical Characterization: Anthocyanins, Phenolics, and Antioxidant Capacity

The biochemical traits of five blueberry varieties, focusing on total anthocyanins, total phenolics, and antioxidant capacity were also examined. Measurements were conducted at both harvest and after postharvest storage, allowing assessment of varietal differences and the stability of key functional compounds over time. These data provide insights into the capacity of each cultivar to retain bioactive compounds, which are important for both nutritional quality and shelf-life.

**Figure 1:**
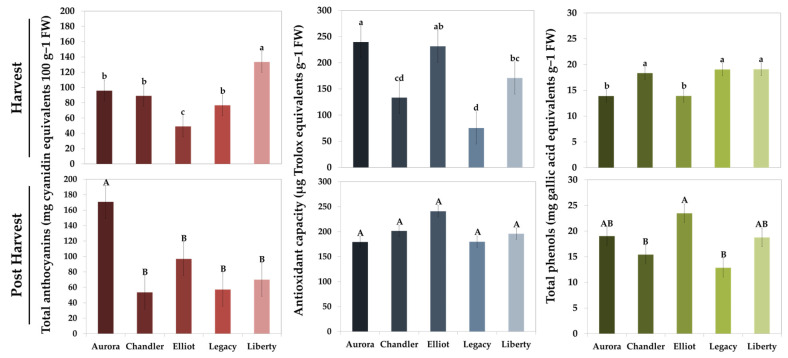
Total anthocyanins [mg cyanidin equivalents 100 g^−1^ fresh weight (FW)], antioxidant capacity [μg Trolox equivalents g^−1^ (FW)], and total phenolics [mg gallic acid equivalents g^−1^ (FW)] of five blueberry (*Vaccinium corymbosum* L.) cultivars (‘Aurora’, ‘Chandler’, ‘Elliot’, ‘Legacy’, and ‘Liberty’) at harvest and postharvest (15 days at 4 °C and 90–95% relative humidity). Values represent mean ± standard error (*n* = 3, with 10 subsamples each). Different lowercase letters indicate significant differences among cultivars at harvest, while different uppercase letters indicate significant differences among cultivars at postharvest, according to Duncan’s Multiple Range Test at *p* ≤ 0.05.

As presented in Figure 1, significant variations in biochemical parameters were observed among the five blueberry cultivars at both the freshly harvested and post-harvest stages. The biochemical parameters assessed included total anthocyanins, total phenolics, and antioxidant capacity. At the harvest stage, ‘Liberty’ exhibited the highest total anthocyanin content (~135 mg 100 g^−1^ fresh weight, FW), significantly surpassing the other cultivars, while ‘Elliot’ showed the lowest value (~50 mg 100 g^−1^ FW). Following harvest, the anthocyanin profile changed in most cultivars, with ‘Aurora’ exhibiting the highest levels postharvest (~170 mg 100 g^−1^ FW).

Regarding antioxidant capacity, ‘Aurora’ displayed the highest value at harvest (~240 μg Trolox equivalents g^−1^ FW), differing significantly from ‘Chandler’, ‘Legacy’, and ‘Liberty’, while ‘Legacy’ exhibited the lowest capacity (~70 μg Trolox equivalents g^−1^ FW).

Variations in total phenolic content among cultivars were less pronounced. At harvest, ‘Chandler’, ‘Legacy’, and ‘Liberty’ exhibited similar levels (~17–20 mg g^−1^ FW). Following cold storage (postharvest stage), ‘Elliot’ reached the highest phenolic content, which differed significantly from that of ‘Chandler’ and ‘Legacy’.

### 3.4. Primary Metabolite Profiles and Cultivar Differentiation

#### 3.4.1. Metabolite Composition Across Cultivars and Storage Stages

**Figure 2:**
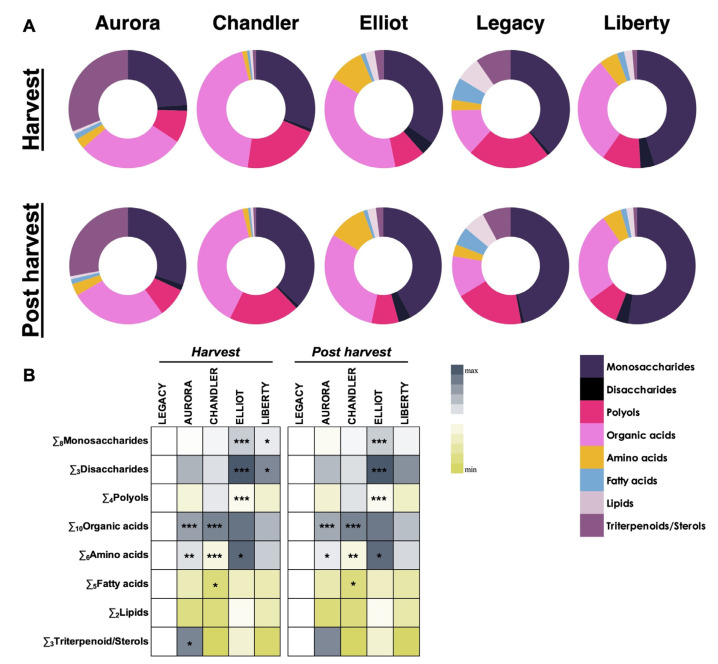
Relative distribution of major metabolite categories, including monosaccharides, disaccharides, polyols, organic acids, amino acids, fatty acids, lipids, and triterpenoids/sterols, in five blueberry (*Vaccinium corymbosum* L.) cultivars (‘Aurora’, ‘Chandler’, ‘Elliot’, ‘Legacy’, and ‘Liberty’) at harvest and after cold storage (15 days at 4 °C and 90–95% relative humidity). Donut charts show the proportional composition of metabolite groups for each cultivar (**A**), while heatmaps illustrate varietal differentiation and compositional shifts between the two stages (**B**). ‘Legacy’ served as the reference cultivar, with grey shading representing relative increases and yellow shading indicating decreases in metabolite concentrations. Color intensity reflects the normalized relative abundance of each metabolite class. Values are expressed as normalized means ± standard error (*n* = 3, with 10 subsamples each). Asterisks in the heatmaps indicate significant differences among cultivars according to the LSD test at * *p* < 0.05, ** *p* < 0.01, and *** *p* < 0.001. Metabolite abundances quantified relative to the internal standard (adonitol) are provided in Supplementary Table S2 to complement the relative distributions shown here.

The distribution and relative abundance of major metabolite classes detected in five blueberry cultivars at harvest and after cold storage (15 days at 4 °C; postharvest stage) are summarized in Figure 2. The proportional contributions of metabolite categories, monosaccharides, disaccharides, polyols, organic acids, amino acids, fatty acids, lipids, and triterpenoids/sterols, highlight cultivar-specific differences and storage-related changes (Figure 2A,B).

At harvest, distinct metabolite profiles were observed among the cultivars. ‘Aurora’ and ‘Chandler’ exhibited significantly higher proportions of organic acids (*p* < 0.001). Amino acid levels varied markedly, being highest in ‘Elliot’ (*p* < 0.05) and lowest in ‘Chandler’ (*p* < 0.001). ‘Elliot’ displayed elevated monosaccharide and disaccharide contents, along with reduced polyols, whereas ‘Liberty’ presented significantly higher monosaccharide and disaccharide proportions relative to the cultivar ‘Legacy’ (*p* < 0.05; Figure 2B). These findings are consistent with previous reports identifying carbohydrates and organic acids as key contributors to blueberry flavor and metabolic differentiation among cultivars.

Following cold storage (postharvest stage), cultivar-dependent changes were evident. ‘Aurora’ largely maintained high concentrations of organic acids (*p* < 0.001) and amino acids (*p* < 0.01), while ‘Chandler’ showed reduced amino acid (*p* < 0.001) and fatty acid (*p* < 0.05) contents. Organic acid levels in ‘Chandler’ remained similar to those at harvest. ‘Elliot’ retained a metabolic composition comparable to its harvest profile, and ‘Liberty’ displayed no significant differences from ‘Legacy’ (Figure 2B).

Overall, these results demonstrate clear cultivar-dependent differences in both the composition and stability of primary metabolites during storage. Variations were particularly pronounced in carbohydrate, organic acid, and amino acid contents, reflecting distinct metabolic patterns associated with each cultivar at harvest and after cold storage (postharvest stage).

#### 3.4.2. Metabolite Profiling and Cultivar-Specific Patterns

**Figure 3:**
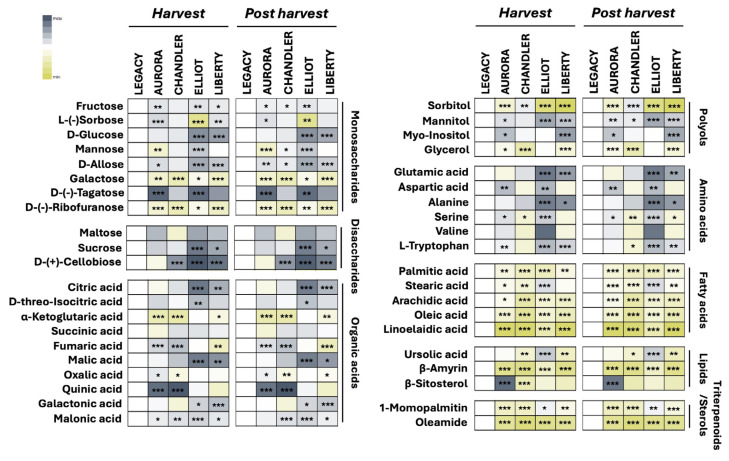
Relative abundance of metabolites in five blueberry (*Vaccinium corymbosum* L.) cultivars (‘Aurora’, ‘Chandler’, ‘Elliot’, ‘Legacy’, and ‘Liberty’) at harvest and after cold storage (15 days at 4 °C and 90–95% relative humidity). The heatmap illustrates varietal differentiation and compositional changes between the two stages. ‘Legacy’ served as the reference cultivar because it represents a widely cultivated highbush blueberry cultivar and exhibited intermediate metabolic levels among the studied cultivars, with grey shading representing relative increases and yellow shading indicating decreases in metabolite concentrations. Color intensity reflects the normalized relative abundance of each metabolite. Values are expressed as means ± standard error (*n* = 3, with 10 subsamples each). Asterisks in the heatmaps indicate significant differences among cultivars (* *p* < 0.05; ** *p* < 0.01; *** *p* < 0.001) according to the LSD test (*p* ≤ 0.05).

Comprehensive metabolite profiling was conducted to explore cultivar-specific metabolic diversity and postharvest alterations (Figure 3, Supplementary Table S2). The heatmap illustrates relative metabolite abundances across cultivars and storage stages, encompassing carbohydrates (monosaccharides, disaccharides, and polyols), organic acids, amino acids, fatty acids, lipids, sterols, and triterpenoids, thereby allowing identification of cultivar-specific metabolic fingerprints.

Distinct accumulation patterns were evident among the five cultivars (‘Aurora’, ‘Chandler’, ‘Elliot’, ‘Legacy’, and ‘Liberty’) at both harvest and postharvest stages.

At harvest, ‘Liberty’ and ‘Elliot’ showed pronounced increases in key monosaccharides, including D-glucose and D-allose, whereas ‘Aurora’ exhibited mixed patterns with significant reductions in mannose, galactose, and D-(–)-ribofuranose. Notably, D-(–)-ribofuranose and galactose were consistently lower in all cultivars relative to ‘Legacy’. Disaccharide profiles revealed higher sucrose levels in ‘Elliot’ and ‘Liberty’, while ‘Chandler’ exhibited elevated cellobiose content. Among polyols, ‘Aurora’, ‘Elliot’, and ‘Liberty’ accumulated higher sorbitol and glycerol levels, whereas ‘Chandler’ showed a significant increase in glycerol but a reduction in sorbitol.

Regarding organic acids, citric and malic acids were elevated in ‘Elliot’ and ‘Liberty’, whereas quinic acid concentrations were higher in ‘Aurora’ and ‘Chandler’, accompanied by suppressed α-ketoglutaric acid levels. For amino acids, ‘Elliot’ exhibited decreased levels of glutamic acid, aspartic acid, alanine, serine, and L-tryptophan, while other cultivars displayed mixed responses. Among fatty acids, lipids, triterpenoids, and sterols, overall metabolite depression was evident, except for increases in stearic acid, ursolic acid, and 1-monopalmitin (‘Elliot’) and β-sitosterol (‘Aurora’).

At the postharvest stage, the overall metabolomic patterns remained largely stable across cultivars, suggesting that cultivar-dependent metabolic traits dominate over maturity-dependent changes in blueberry fruits. These findings indicate that both cultivar and ripening stage critically influence metabolic composition, with direct implications for postharvest quality and targeted nutritional selection in breeding programs.

### 3.5. Volatile Organic Compounds at Harvest: Quantification and Profiling

#### 3.5.1. Volatile Compound Composition at Harvest

At the commercial harvest stage, when aroma expression reaches its maximum intensity, the total volatile compounds were identified and quantified across five blueberry cultivars. A total of 39 volatiles were detected and classified into seven major chemical groups: sulfides, aldehydes, ketones, acids, phenols, alcohols, and esters. These volatiles are key contributors to the characteristic aroma and overall sensory quality of blueberries.

**Figure 4:**
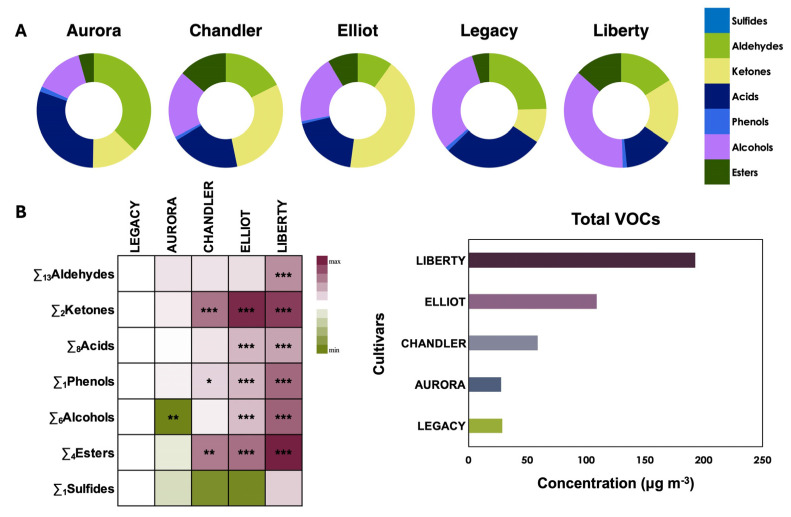
Relative abundance of major volatile compound classes, sulfides, aldehydes, ketones, acids, phenols, alcohols, and esters, in five blueberry (*Vaccinium corymbosum* L.) cultivars (‘Aurora’, ‘Chandler’, ‘Elliot’, ‘Legacy’, and ‘Liberty’) at the commercial harvest stage. Donut charts show the proportional distribution of each volatile group per cultivar (**A**), while the heatmap illustrates varietal differentiation and compositional variability (**B**). ‘Legacy’ served as the reference cultivar, with red shading representing relative increases and green shading indicating decreases in metabolite concentrations. Color intensity reflects the normalized relative abundance of each metabolite. Values are expressed as means ± standard error (*n* = 3, with 10 subsamples each). Asterisks in the heatmaps indicate significant differences among cultivars (* *p* < 0.05; ** *p* < 0.01; *** *p* < 0.001) according to the LSD test (*p* ≤ 0.05).

As shown in Figure 4 and Supplementary Table S3, distinct cultivar-dependent differences were evident in the composition and relative abundance of volatile compounds. ‘Liberty’, ‘Elliot’, and ‘Chandler’ exhibited significantly higher levels of ketones (*p* < 0.001), phenols (‘Liberty’, ‘Elliot’, *p* < 0.001; ‘Chandler’, *p* < 0.05), and esters (‘Liberty’, ‘Elliot’, *p* < 0.001; ‘Chandler’, *p* < 0.001). In addition, ‘Liberty’ and ‘Elliot’ presented elevated concentrations of acids [(20.8 μg m^−3^) and (26.7 μg m^−3^)] (*p* < 0.001) and total alcohols [21.2 μg m^−3^ (‘Elliot’) and 71.2 μg m^−3^ (‘Liberty’)] (*p* < 0.001), whereas ‘Aurora’ (3.8 μg m^−3^) showed a significant reduction in alcohols (*p* < 0.01). Moreover, ‘Liberty’ (30.9 μg m^−3^) had increased aldehyde concentrations (*p* < 0.001) compared with the reference cultivar ‘Legacy’ (6.9 μg m^−3^) (Supplementary Table S3).

These findings reveal substantial biochemical variation among cultivars in volatile composition, reflecting differences in their inherent aromatic potential. Understanding these cultivar-specific volatile profiles provides valuable insights for breeding programs and postharvest quality management strategies aimed at optimizing blueberry flavor and consumer appeal.

#### 3.5.2. Cultivar-Specific Volatile Profiles

**Figure 5:**
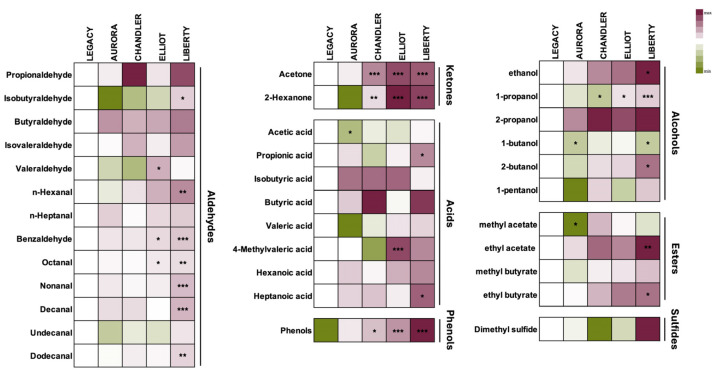
Relative abundance of volatile organic compounds in five blueberry (*Vaccinium corymbosum* L.) cultivars (‘Aurora’, ‘Chandler’, ‘Elliot’, ‘Legacy’, and ‘Liberty’) at harvest. The heatmap illustrates varietal differentiation and compositional variability among cultivars. ‘Legacy’ served as the reference cultivar because it represents a widely cultivated cultivar with relatively balanced metabolite levels among the studied cultivars, with purple shading representing relative increases and green shading indicating decreases in metabolite concentrations. Color intensity reflects the normalized relative abundance of each metabolite. Values are expressed as means ± standard error (*n* = 3, with 10 subsamples each). Asterisks in the heatmaps indicate significant differences among cultivars (* *p* < 0.05; ** *p* < 0.01; *** *p* < 0.001) according to the LSD test (*p* ≤ 0.05).

Distinct accumulation patterns were evident among the five cultivars (‘Aurora’, ‘Chandler’, ‘Elliot’, ‘Legacy’, and ‘Liberty’) at harvest. ‘Legacy’ served as the reference cultivar, with purple shading representing relative increases and green shading indicating decreases in volatile concentrations (Figure 5).

‘Liberty’ exhibited markedly higher abundances of aldehydes, including isobutyraldehyde, n-hexanal, benzaldehyde, octanal, nonanal, decanal, and dodecanal, while ‘Elliot’ showed significant increases in valeraldehyde, benzaldehyde, and octanal (*p* < 0.05). Among ketones, acetone and 2-hexanone were highly accumulated in ‘Liberty’, ‘Elliot’, and ‘Chandler’.

Regarding organic acids, ‘Elliot’ showed a strong accumulation of 4-methylvaleric acid (*p* < 0.001), whereas ‘Liberty’ displayed elevated levels of propionic and heptanoic acids (*p* < 0.05). Conversely, acetic acid concentrations were significantly lower in ‘Aurora’ (*p* < 0.05).

Phenolic volatiles were also elevated in ‘Liberty’ (*p* < 0.001), ‘Elliot’ (*p* < 0.01), and ‘Chandler’ (*p* < 0.05), highlighting clear cultivar-specific differentiation. Similarly, alcohol accumulation was highest in ‘Liberty’, characterized by increases in 1-propanol (*p* < 0.001), ethanol (*p* < 0.05), and 2-butanol (*p* < 0.05), while 1-butanol was significantly reduced in both ‘Liberty’ and ‘Aurora’ (*p* < 0.05).

Finally, esters were notably enriched in ‘Liberty’, with higher concentrations of ethyl acetate (*p* < 0.01) and ethyl butyrate (*p* < 0.05), whereas methyl acetate was significantly suppressed in ‘Aurora’.

Overall, the volatile compound analysis highlights pronounced cultivar-dependent variations that define the aromatic profiles of blueberry fruits. These compositional differences underpin the sensory distinctiveness of each cultivar and provide a biochemical basis for targeted flavor enhancement through cultivar selection and postharvest management.

### 3.6. Principal Component Analysis of Metabolites and Volatiles

**Figure 6:**
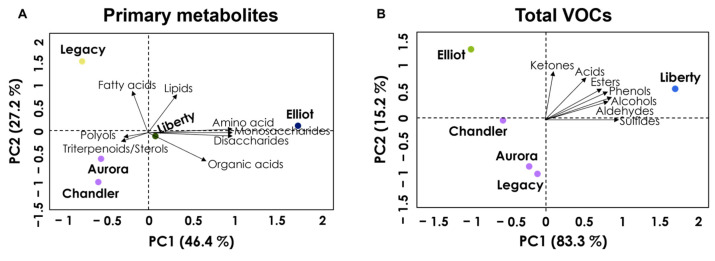
Principal Component Analysis (PCA) score plots showing the distribution of five blueberry (*Vaccinium corymbosum* L.) cultivars (‘Aurora’, ‘Chandler’, ‘Elliot’, ‘Legacy’, and ‘Liberty’) based on datasets obtained at harvest: (**A**) primary metabolites and (**B**) volatile compounds. The first two components (PC1 and PC2) explain 73.6% (**A**) and 98.5% (**B**) of the total variance, highlighting the stronger discriminatory power of volatile compounds for cultivar differentiation. Ellipses represent the 95% confidence intervals for each cultivar group.

Principal Component Analysis (PCA) was conducted to assess the relationships among blueberry cultivars based on their integrated primary metabolite and volatile compound profiles at harvest stage. The PCA score plots (Figure 6) revealed distinct clustering patterns.

For primary metabolites (Figure 6A), the first two components explained 73.6% of the total variance (PC1: 46.4%, PC2: 27.2%), separating cultivars primarily based on their organic acid versus sugar/amino acid balances. For volatile compounds (Figure 6B), the differentiation was even more pronounced, with the first two components accounting for 98.5% of the variance (PC1: 83.3%, PC2: 15.2%). PC1 distinctly separated ‘Liberty’ from the other cultivars, driven by its high relative abundance of aldehydes and alcohols. The variables with the highest loadings on PC1 mainly belonged to aldehydes, alcohols, phenols, and sulfur-containing compounds, suggesting that cultivar separation along this axis reflects differences in key aroma-related metabolic pathways. In particular, aldehydes and alcohols are commonly associated with fresh/green and fruity notes, whereas phenolic and sulfur-containing volatiles may contribute to more intense and distinctive aroma characteristics.

Overall, the PCA confirms that while primary metabolites reveal metabolic nuances, volatile signatures provide the strongest discriminative power for these blueberry cultivars.

## 4. Discussion

This study provides an integrated evaluation of the physicochemical, biochemical, and metabolomic attributes of five highbush blueberry (*Vaccinium corymbosum* L.) cultivars. The selection included cultivars with diverse parental backgrounds, which accounts for the wide variance observed in their metabolic stability. By linking compositional diversity with postharvest quality, the results confirm that cultivar-specific factors are the dominant determinants of fruit quality. This inherent biological identity dictates the balance of sugars and acids, the profile of bioactive compounds, and the unique volatile signatures of each cultivar [9,25,26,27].

### 4.1. Color and Physical Quality

Color parameters (L*, a*, b*, C*, and *h*°) varied significantly among cultivars, reflecting inherent differences in pigmentation and surface characteristics. While such variations could be interpreted as differences in ripening stages, all samples in this study were harvested at a uniform commercial maturity (fully blue). Therefore, these differences reflect cultivar-specific metabolic characteristics and genetic variation in fruit surface properties. ‘Chandler’ exhibited the highest C* values relative to the other cultivars evaluated, whereas ‘Liberty’ was more prone to hue angle shifts. These findings suggest that cultivar influence visual quality, which is a critical determinant of consumer preference [9]. However, the most desirable color attributes may vary depending on market standards and consumer expectations, and higher C* values (color saturation) do not necessarily correspond to greater consumer acceptance.

Although no correlation analysis was performed between anthocyanin content and color parameters, changes in values may reflect differences in redness that are often associated with anthocyanin accumulation. ‘Chandler’ consistently exhibited high Chroma (C*) values, indicating a more saturated color, whereas ‘Liberty’ was more prone to hue angle shifts, reflecting cultivar-specific color dynamics during maturation. Physically, textural integrity did not correlate strictly with fruit size; ‘Chandler’ produced the largest fruits, yet ‘Legacy’ and ‘Liberty’ exhibited superior firmness. This uncoupling of size and firmness suggests that cell wall architecture and turgor pressure maintenance are genetically distinct traits, as supported by recent genomic studies [8,9].

### 4.2. Biochemical Characteristics and Antioxidant Retention

Anthocyanin content and antioxidant capacity revealed distinct cultivar-dependent stability. ‘Liberty’ showed the highest anthocyanin levels at harvest but experienced a marked decline after cold storage (postharvest stage). In contrast, ‘Aurora’ retained high anthocyanin stability and antioxidant capacity. While individual anthocyanins were not quantified in this study, this differential stability may potentially be linked to the specific anthocyanin profile. It has been suggested that differences in the relative abundance of individual, non-acylated, anthocyanins among cultivars may influence pigment stability during storage [28,29,30], providing a metabolic basis for the high retention observed in ‘Aurora’. Although methodological factors can contribute to variability, the strong cultivar-dependent post-storage shifts observed here are consistent with previous blueberry studies reporting heterogeneous anthocyanin responses across cultivars. Recent research showed that total anthocyanins during postharvest storage may follow various trends depending on genotype, indicating that large differences after storage can be expected even under controlled conditions [31]. Therefore, the fluctuations observed in this study likely reflect inherent biological differences in postharvest anthocyanin stability among the investigated cultivars.

### 4.3. Primary Metabolites and the Chemical Basis of Flavor Potential

The metabolomic profiling revealed that differences in flavor potential are driven by specific shifts in primary carbon metabolism. ‘Legacy’ exhibited the highest SSC/TA ratio (19.7), a parameter widely regarded in the literature as a key indicator of consumer preference [32]. Metabolically, this potential flavor profile was not driven only by superior sugar accumulation, as ‘Liberty’ and ‘Elliot’ possessed higher relative abundances of glucose, fructose, and sucrose (Figure 3), but rather by a significantly lower accumulation of organic acids, resulting in lower titratable acidity (Table 2).

Conversely, the specific organic acid profile played a defining role in cultivar differentiation. ‘Aurora’ was characterized by high acidity, which metabolically correlated with elevated levels of quinic and fumaric acids (Figure 3). The accumulation of quinic acid, often associated with a sharper or more astringent taste profile, may reflect an increased contribution of the shikimate pathway [33,34]. In contrast, the acidity in ‘Elliot’ and ‘Liberty’ was driven primarily by citric and malic acids (Figure 3), which typically contribute to a fresher perception of sourness [35]. Consequently, ‘Liberty’ presented a metabolically intense profile characterized by both high sugars and high citric/malic acids, suggesting a complex, full-bodied flavor, whereas ‘Legacy’ achieved its flavor profile through the lack of competitive acidity.

### 4.4. Metabolic Trade-Offs and Carbon Partitioning

An integrated view of the metabolic data may suggest a potential trade-off in carbon partitioning between cultivars, as supported by the multivariate relationships observed in the PCA (Figure 6). ‘Liberty’ and ‘Elliot’ appeared to prioritize carbon allocation towards carbohydrate accumulation (i.e., high monosaccharides) and volatile synthesis (i.e., high esters/aldehydes), traits often associated with sensory appeal and immediate sensory appeal [36]. In contrast, ‘Aurora’ exhibited a metabolic profile skewed towards organic acids (i.e., quinic, fumaric) and secondary metabolites (i.e., anthocyanins, antioxidants). Since quinic acid is a key precursor in the shikimate pathway, which feeds into the phenylpropanoid pathway responsible for anthocyanin biosynthesis, the high levels of both traits in ‘Aurora’ may be consistent with a stronger metabolic flux through this pathway [27]. These observations may reflect potential differences in carbon allocation strategies among cultivars; however, such interpretations remain speculative. In this context, ‘Aurora’ may exhibit metabolic characteristics associated with enhanced antioxidant-related metabolism, whereas ‘Liberty’ may display a metabolic profile favoring sweetness and aroma biosynthesis. However, this proposed trade-off remains an interpretive hypothesis based on multivariate metabolomic patterns and should be validated through further research, including transcriptomic approaches targeting key regulatory genes involved in carbon allocation, the shikimate/phenylpropanoid pathway, and VOC biosynthesis.

### 4.5. Volatile Compounds and Aroma Complexity

Volatile profiling provided the strongest discrimination among cultivars, confirming that aroma is the most variable component of the blueberry phenotype. ‘Liberty’ displayed a complex “fruity” and “sweet” volatile signature, driven by the high coexistence of esters (ethyl acetate, ethyl butyrate) and aldehydes (i.e., nonanal, decanal). Esters are synthesized via the catabolism of fatty acids and amino acids, and their elevation in ‘Liberty’ correlates with the specific lipid and amino acid precursors identified in the primary metabolite analysis [37,38,39]. Conversely, ‘Aurora’ showed a lower relative abundance of alcohols and esters, which may align with a lower aromatic intensity based on its VOC profile. The distinct accumulation of specific marker compounds, such as 1-propanol in ‘Liberty’ and 4-methylvaleric acid in ‘Elliot’, may suggest cultivar-specific regulation of the lipoxygenase (LOX) and amino acid degradation pathways [6,12]. These volatile fingerprints are critical, as they modulate the perception of sweetness beyond what is explained by sugar content alone [40,41]. Finally, regarding the volatile profile, this study prioritized the characterization of the distinct aromatic fingerprints at the commercial harvest stage, representing the maximum aromatic potential of each cultivar. Therefore, VOC analysis was performed only at harvest. While post-harvest storage is known to alter volatile composition, quantifying these specific degradation pathways was considered outside the scope of the present cultivar-focused evaluation and warrants a dedicated longitudinal investigation [7,42].

### 4.6. Integrated Metabolic Signatures

The Principal Component Analysis (PCA) provided an integrative multivariate overview by reducing dataset dimensionality and highlighting the variables contributing most strongly to cultivar discrimination. PCA results reinforced that while primary metabolites provide the “body” of the fruit flavor (sweetness and acidity), volatile compounds provide the “identity” [43,44,45]. The clear separation of ‘Liberty’ along PC1 (Figure 6) was driven almost exclusively by its diverse volatile profile, whereas ‘Aurora’ and ‘Chandler’ clustered based on their organic acid-rich primary metabolism. This pattern complements the individual metabolite and VOC analyses by confirming that volatile signatures are the primary drivers of cultivar separation, while primary metabolites mainly shape differences in sweetness–acidity balance. This dichotomy illustrates that breeding for high sugars (SSC) alone is insufficient for superior quality; optimizing the specific volatile background is essential for enhancing the perceived flavor complexity. Nevertheless, these mechanistic interpretations remain hypothetical and require validation through further systems biology approaches, such as transcriptomic and proteomic analyses.

## 5. Concluding Remarks

In conclusion, this integrated metabolomic and physicochemical assessment demonstrates that blueberry (*Vaccinium corymbosum* L.) fruit quality is governed by a tightly regulated network of primary and secondary metabolites that is fundamentally cultivar-dependent. While primary metabolites, such as organic acids and sugars, establish the basic sweetness–acidity balance, it is the volatile organic compounds (VOCs) that serve as the primary discriminators of cultivar identity. This study highlights that a high soluble solid content (SSC) does not inherently guarantee a balanced flavor profile unless coupled with appropriate acidity and aromatic complexity, as evidenced by the superior SSC/TA ratio in ‘Legacy’ driven by low acid accumulation.

A central finding of this research is the differential stability of anthocyanins and antioxidants during cold storage (postharvest stage), which emphasizes that bioactive retention is as variable and cultivar-dependent as sensory quality. Our results suggest a metabolic trade-off between immediate sensory appeal and long-term functional value. Cultivars such as ‘Liberty’ provide exceptional aromatic profiles and high sugar/acid intensity ideal for immediate fresh consumption, whereas ‘Aurora’ and ‘Elliot’ demonstrate superior bioactive retention, making them the most suitable candidates for prolonged cold storage (postharvest stage) and the maintenance of health-promoting properties.

These insights provide a robust scientific basis for targeted breeding programs: selection strategies should prioritize specific metabolic markers, such as low quinic acid for sweetness enhancement or specific ester combinations for aromatic complexity, rather than relying on broad agronomic traits. Future research integrating transcriptomic analysis with these metabolic phenotypes will be essential to uncover the specific enzymatic regulators controlling these quality-determining pathways and to further optimize bioactive retention in *Vaccinium* species.

## Figures and Tables

**Table 1:** Color parameters (L*, a*, b*, C*, and *h*°) of five blueberry (*Vaccinium corymbosum* L.) cultivars (‘Aurora’, ‘Chandler’, ‘Elliot’, ‘Legacy’, and ‘Liberty’) measured at harvest and after cold storage (15 days at 4 °C and 90–95% relative humidity).

	Harvest																			
	Lightness (L*)				Redness (a*)				Yellowness (b*)				Chroma (C*)				Hue Angle (*h*^o^)			
**Aurora**	11.8	±	0.4	a	7.0	±	0.7	b	0.1	±	0.1	b	7.0	±	0.7	b	226.1	±	31.4	a
**Chandler**	11.3	±	0.4	a	10.5	±	1.1	a	1.1	±	0.4	a	10.6	±	1.2	a	134.9	±	31.5	b
**Elliot**	11.2	±	0.4	a	4.7	±	0.8	b	0.0	±	0.2	b	4.8	±	0.8	b	269.8	±	27.2	a
**Legacy**	12.4	±	0.5	a	4.8	±	0.7	b	−0.2	±	0.2	b	4.9	±	0.7	b	256.0	±	28.1	a
**Liberty**	12.5	±	0.4	a	7.1	±	0.8	b	0.3	±	0.2	b	7.1	±	0.8	b	190.8	±	32.3	ab
	**Post Harvest**																			
	Lightness (L*)				Redness (a*)				Yellowness (b*)				Chroma (C*)				**Hue Angle (*h*^o^)**			
**Aurora**	10.0	±	0.1	C	4.2	±	0.4	B	−0.2	±	0.0	BC	4.3	±	0.4	B	295.5	±	24.5	AB
**Chandler**	10.4	±	0.2	BC	8.4	±	0.7	A	0.5	±	0.2	A	8.4	±	0.7	A	169.1	±	32.7	C
**Elliot**	11.0	±	0.3	AB	2.1	±	0.2	C	−0.4	±	0.1	C	2.2	±	0.2	C	338.1	±	11.7	A
**Legacy**	11.2	±	0.2	A	2.4	±	0.2	C	−0.5	±	0.1	C	2.5	±	0.2	C	347.2	±	1.7	A
**Liberty**	11.8	±	0.4	A	4.6	±	0.4	B	−0.1	±	0.1	B	4.6	±	0.4	B	249.3	±	29.9	B

Values are expressed as the mean ± standard error (*n* = 3, with 10 subsamples each). Different lowercase letters within the same column indicate significant differences among cultivars at harvest, while different uppercase letters within the same column indicate significant differences among cultivars after cold storage (postharvest stage) according to Duncan’s Multiple Range Test at *p* ≤ 0.05.

**Table 2:** Quality characteristics, including fresh weight (g), height (mm), width (mm), firmness (g), soluble solids content (SSC, %), titratable acidity (TA, %), and the SSC/TA ratio, of five blueberry (*Vaccinium corymbosum* L.) cultivars (‘Aurora’, ‘Chandler’, ‘Elliot’, ‘Legacy’, and ‘Liberty’) evaluated at harvest (Day 0) and after cold storage (15 days at 4 °C and 90–95% relative humidity).

	**Harvest**																											
	**Fresh Weight (g)**				**Height (mm)**				**Width (mm)**				**Firmness (g)**				**SSC (%)**				**TA (%)**				**SSC/TA**			
**Aurora**	1.43	±	0.0	b	10.1	±	0.2	b	14.4	±	0.2	b	62.8	±	2.1	a	12.0	±	0.3	b	2.1	±	0.1	a	5.8	±	0.3	c
**Chandler**	1.74	±	0.1	a	11.4	±	0.2	a	15.4	±	0.2	a	63.8	±	2.6	a	12.4	±	0.4	b	1.1	±	0.2	bc	12.0	±	2.1	b
**Elliot**	1.24	±	0.0	c	10.0	±	0.1	b	13.6	±	0.2	c	56.2	±	2.0	b	11.5	±	0.5	b	1.4	±	0.1	b	8.1	±	1.0	bc
**Legacy**	1.14	±	0.1	c	10.2	±	0.2	b	12.8	±	0.2	d	68.2	±	2.4	a	14.2	±	0.4	a	0.7	±	0.1	c	19.7	±	1.7	a
**Liberty**	1.13	±	0.0	c	9.2	±	0.1	c	13.3	±	0.2	cd	66.7	±	1.3	a	12.6	±	0.4	b	1.3	±	0.2	b	10.0	±	1.8	bc
	**Post Harvest**																											
	**Fresh Weight (g)**				**Height (mm)**				**Width (mm)**				**Firmness (g)**				**SSC (%)**				**TA (%)**				**SSC/TA**			
**Aurora**	1.45	±	0.0	B	10.2	±	0.1	BC	14.9	±	0.1	B	55.3	±	2.1	C	12.2	±	0.4	A	1.7	±	0.2	A	7.2	±	1.0	C
**Chandler**	1.85	±	0.1	A	11.6	±	0.1	A	15.9	±	0.2	A	56.5	±	1.6	C	11.0	±	0.1	B	0.9	±	0.0	C	12.2	±	0.3	AB
**Elliot**	1.26	±	0.0	C	10.0	±	0.1	C	14.2	±	0.2	C	60.8	±	2.1	BC	10.7	±	0.4	B	1.5	±	0.2	AB	7.6	±	1.2	BC
**Legacy**	1.15	±	0.0	C	10.5	±	0.1	B	13.7	±	0.2	C	66.2	±	2.9	AB	12.4	±	0.4	A	0.8	±	0.1	C	16.7	±	2.6	A
**Liberty**	1.16	±	0.0	C	9.4	±	0.1	D	13.5	±	0.4	C	67.8	±	1.7	A	12.5	±	0.3	A	1.1	±	0.1	BC	11.1	±	1.0	BC

Values are expressed as the mean ± standard error (*n* = 3, with 10 subsamples each). Different lowercase letters within the same column indicate significant differences among cultivars at harvest, while different uppercase letters within the same column indicate significant differences among cultivars after cold storage (postharvest stage), according to Duncan’s Multiple Range Test at *p* ≤ 0.05.

## Data Availability

The datasets generated and analyzed during the current study are available from the corresponding author upon reasonable request due to privacy concerns.

## Supplementary Materials

The following supporting information can be downloaded at https://www.mdpi.com/article/10.3390/plants15060948/s1, Table S1: Calibration parameters and method validation data for target compounds; Table S2: Relative abundance of primary metabolites in five blueberry cultivars (‘Aurora’, ‘Chandler’, ‘Elliot’, ‘Legacy’, and ‘Liberty’) at harvest and after cold storage (postharvest stage). Values are expressed as mean ± standard error (SE) (n = 3, with 10 subsamples each). Different lowercase letters within the same row indicate significant differences among cultivars at harvest, while different uppercase letters within the same row indicate significant differences among cultivars after cold storage (postharvest stage), according to Duncan’s Multiple Range Test at *p* ≤ 0.05. Metabolite levels were quantified relative to the internal standard (adonitol); Table S3: Concentrations (μg m^−3^) of volatile organic compounds in five blueberry cultivars (‘Aurora’, ‘Chandler’, ‘Elliot’, ‘Legacy’, and ‘Liberty’) at harvest. Values are presented as mean ± standard error (SE) (n = 3, with 10 subsamples each). Different letters within the same row indicate significant differences among cultivars at harvest, according to Duncan’s Multiple Range Test at *p* ≤ 0.05.
